# A Cone-Beam Computed Tomography-Based Assessment of Safe Zones for Orthodontic Mini-Implant Placement in the Lateral Maxilla: A Retrospective Morphometric Study

**DOI:** 10.3390/diagnostics15101252

**Published:** 2025-05-15

**Authors:** Iva Jakovljevic, Pavle Milanovic, Milica Vasiljevic, Jovana Milanovic, Momir Z. Stevanovic, Nemanja Jovicic, Milos Stepovic, Vladimir Ristic, Dragica Selakovic, Gvozden Rosic, Aleksandra Arnaut

**Affiliations:** 1 Department of Dentistry, Faculty of Medical Sciences, University of Kragujevac, 34000 Kragujevac, Serbia; iva_obradovic@yahoo.com (I.J.); pavle11@yahoo.com (P.M.); milicavaska13@gmail.com (M.V.); jovannakg94@gmail.com (J.M.); momirstevanovic7@gmail.com (M.Z.S.); vristic7@gmail.com (V.R.); sandra11_92@yahoo.com (A.A.); 2 Department of Histology and Embryology, Faculty of Medical Sciences, University of Kragujevac, 34000 Kragujevac, Serbia; nemanjajovicic.kg@gmail.com; 3 Department of Anatomy, Faculty of Medical Sciences, University of Kragujevac, 34000 Kragujevac, Serbia; stepovicmilos@yahoo.com; 4 Department of Physiology, Faculty of Medical Sciences, University of Kragujevac, 34000 Kragujevac, Serbia; dragica984@gmail.com

**Keywords:** orthodontic mini-implant, CBCT, maxilla, safe zones, temporary anchorage devices

## Abstract

**Background/Objectives:** Orthodontic temporary anchorage devices (TADs) in the lateral maxillary region are useful tools for successful orthodontic treatment. Radiological anatomical knowledge is crucial for the successful placement of TADs. The use of cone-beam computed tomography (CBCT) is essential for evaluating the relationship between the ideal placement point (IPP) and dental structures, particularly in cases with anatomical limitations. Accordingly, this study aims to assess the anatomical conditions for orthodontic mini-implant (MI) insertion in the posterior maxilla using CBCT as the gold standard. **Methods:** This retrospective study included 62 patients (37.1% male, 62.9% female) aged 11 to 50 years. CBCT scans (sagittal and axial cross-sections) were used to evaluate interdental bone characteristics in different regions. The evaluated regions were defined as follows: Region 1 (canine and first premolar), Region 2 (first and second premolars), Region 3 (second premolar and first molar), and Region 4 (first and second molars). All parameters were assessed at three predefined levels: A, B, and C, located 4, 3, and 2 mm, respectively, from the alveolar crest. At the aforementioned levels, we performed measurements, such as the interdental width (IDW) in the mesiodistal direction and buccopalatal depth (BPD). The last observation was the relationship between the ideal TAD placement point (IPP) and dental structures, such as contact points (CPs) and cusp tips (C1-cusp of mesial tooth, C2-cusp of distal tooth, in each region). **Results:** A statistically significant positive correlation was found between the IDW and BPD at Levels A, B, and C in Region 1, while a negative correlation was observed between the IDW and BPD at Level C in Region 2′. The highest percentages of IDW exceeding 3 mm were found in Region 4 at Level A (67.7%), followed by Region 1′ and 2′, both at Level A. The mean interdental width measured at each level on the right and left sides was highest at Level A, exceeding 3 mm, and the width decreased with each successive level. The mean BPD measured at each level on the right and left sides was also highest at Level A. **Conclusions:** This methodological approach could assist in ensuring precise and efficient implant insertion. Furthermore, it can be concluded that the safe zone for buccal and interdental mini-implant placement is located 4 mm from the alveolar crest at Level A. Also, the CBCT analysis algorithm may serve as a valuable tool for clinicians in determining optimal TAD placement in different dental regions.

## 1. Introduction

Temporary anchorage devices (TADs, orthodontic mini-implants [MIs], mini-screws) are a part of modern orthodontics. Their application allows orthodontists to treat patients with greater accuracy and fewer complications, contributing to better final outcomes and reduced treatment time [1,2]. There are numerous indications for their use, such as dental and skeletal movements [2]. Furthermore, in complex cases with a potential loss of anchorage, MIs could prevent the least favorable orthodontic treatment outcome [3]. Based on the current literature [4], mini-implants used for increasing anchorage are most commonly placed in the buccal interdental bone. They offer advantages such as small size, low cost, streamlined insertion and removal procedures, and immediate load while providing adequate anchorage to enable orthodontic movements [5,6].

The use of orthodontic MIs can sometimes lead to certain complications, which may include inflammation and infection of the surgical site, soft tissue irritation, implant mobility or failure, damage to adjacent structures, pain and discomfort, bone loss, and other complications [7,8,9]. In order to prevent the aforementioned complications, the location of the MI insertion should be determined based on the patient’s anatomical characteristics. [10] If the amount of interdental bone width, the buccopalatal depth, and the inclination of the teeth are not accurately assessed, the risk of complications increases [10,11,12].

There are many protocols for selecting the site for mini-implant placement [13,14]. In cases that require MI insertion in the buccal plate, the guidelines are as follows: the MI should be inserted into the interdental bone, ensuring it is 1 mm away from the surrounding anatomical structures. This includes maintaining 1 mm from the alveolar crest, 1 mm from the tooth root, and 1 mm from the mucogingival border [8]. Another anatomical characteristic that should be taken into consideration during mini-implant placement is the soft tissue. MIs placed in mobile soft tissue areas have experienced failures, with an increased risk of tissue irritation and inflammation [15]. The attached gingiva is 5 mm above the alveolar bone crest, and this defines the upper limit for safe implant placement [16]. Data from the current literature reveal that guidelines for selecting the mini-implant insertion point are determined based on the aforementioned anatomical structures. However, the distance of this point relative to clinically visible surrounding anatomical structures has not been analyzed to date.

Due to the specificity and small surgical area, it is not surprising that MIs offer different macrodesign features, which refer to the structural features that enhance the stability and ease of placement of MIs within the bone [3]. Key aspects of the macrodesign include mini-implants typically having a screw-like design with a diameter ranging between 1 and 2 mm [1,8]. MIs are generally shorter than conventional dental implants, with lengths ranging from 6 to 12 mm. The length is selected based on the bone thickness at the implantation site, ensuring sufficient anchorage without causing trauma to deeper structures [8]. TADs are not completely stable, as they tend to shift by an average of less than 0.5 mm. Therefore, it is advisable to maintain a 2 mm space (1 mm on each side) between the mini-screw and the adjacent anatomical structures [16]. The roots of adjacent teeth are also at risk and may be damaged if interdental space is not properly assessed [7,9,15].

The length of the MI does not impact stability for screws longer than 5 mm, whereas screw diameter has shown a significant correlation with stability [10]. Unlike endosseous dental implants, which achieve stability through osseointegration, orthodontic mini-screws rely on mechanical retention and can shift within the bone [11].

To adequately address anatomical requirements and optimize the biomechanical potential of TADs, radiographic evaluation is of critical importance [17]. Namely, OPG is frequently used in orthodontics and dentistry due to its convenience and broad imaging coverage of dental structures. Despite its advantages for initial assessments, OPG has notable limitations in detailed planning for mini-implant (MI) placement because of its two-dimensional (2D) nature, which may result in image distortion and magnification errors [17,18,19,20,21]. These inaccuracies can affect critical measurements, such as interdental space and bone density, necessary for safe MI placement [18,19]. Research by MacDonald et al. [17] demonstrated CBCT’s superiority over OPG for the accurate depiction of buccopalatal dimensions and bone quality, critical for long-term MI stability [12]. In contrast, periapical radiographs are simpler, less expensive, involve lower radiation doses, and can also be used to assess the proximity of the MI to tooth roots [22]. Similarly, as is the case with OPG, they are not useful in registering numerous anatomical structures or measuring interdental spaces in three dimensions, as they cannot register all anatomical structures and interdental spaces in three dimensions [22,23,24].

On the other hand, cone-beam computed tomography (CBCT) has become an essential imaging modality in modern orthodontics due to its ability to provide three-dimensional (3D) visualizations of dental and skeletal structures [24,25]. Unlike traditional two-dimensional radiographs, CBCT offers a more comprehensive view, allowing for the precise measurements of bone density, interdental bone volume, and proximity to vital anatomical structures such as tooth roots and maxillary sinuses [26,27]. This is particularly crucial when planning the placement of orthodontic mini-implants, as the accurate assessment of bone morphometric characteristics can significantly reduce the risk of complications and enhance implant stability [28,29,30,31]. Recent studies have demonstrated that CBCT can effectively identify the optimal insertion sites for MIs by providing detailed cross-sectional images of the buccal and palatal bone regions [30,32]. For instance, Park et al. [33] reported that using CBCT significantly improved the accuracy of mini-implant placement, reducing the incidence of root contact and enhancing primary stability. Also, a few studies [34,35] described the interdental space as a potential TAD site using CBCT. Moreover, CBCT has proven valuable in diagnosing complex cases of malocclusion, offering orthodontists the ability to assess skeletal discrepancies, airway space, and temporomandibular joint conditions with higher precision than conventional methods [24].

Given its enhanced diagnostic capabilities and lower radiation doses compared to traditional CT scans, CBCT is now considered the gold standard for planning some orthodontic procedures. The evaluation of TAD placement sites is especially important in cases with potential iatrogenic damage to surrounding structures and tooth movement in a compromised anatomical configuration (e.g., retraction of maxillary incisors toward the nasopalatine canal) [23]. In order to facilitate MI placement, the combination of CBCT and specific software allows for the creation of high-precision surgical guides. However, this procedure may lead to increased costs, as well as the lack of technical support for planning and guiding fabrication, extending overall treatment duration [36].

Based on the current literature, there is a lack of publications addressing the relationship between interdental septum analysis and dental structures from the perspective of TAD placement using CBCT imaging. Accordingly, the aim of this study is to identify more reliable algorithms that connect anatomical characteristics such as the interdental bone width (IDW), the buccopalatal bone depth (BPD), and the adjacent teeth. This approach may assist clinicians in selecting optimal TAD insertion sites in anatomically compromised regions, thereby reducing the risk of complications.

## 2. Materials and Methods

### 2.1. Study Design

This retrospective study was conducted using the CBCT images of patients treated at the Department of Dentistry, Faculty of Medical Sciences, University of Kragujevac, Serbia, between July 2018 and September 2024. The study protocol received approval from the institutional review board of the Faculty of Medical Sciences, University of Kragujevac (approval ID 01-14697), and was conducted in accordance with the latest version of the Declaration of Helsinki. The patients included in this study underwent 3D imaging of the maxilla for various clinical reasons, such as the assessment of dental anomalies, surgical planning, or other dental treatments. Among them, some required the placement of orthodontic mini-implants, but only those who met the established inclusion criteria were selected for further analysis, ensuring the relevance and reliability of the findings.

The inclusion criteria were as follows: age between 11 and 50 years, complete dentition in the maxillary arch excluding the third molars, high-quality CBCT volumetric data, and verified ethnicity from medical records. Given the growing application of orthodontic mini-implants during the early permanent dentition period and their use in pre-prosthetic orthodontic preparation among older patients, this study was designed to encompass a wide age range of participants [37,38,39,40,41]. This approach ensures a comprehensive understanding of the utility and adaptability of mini-implants across different developmental and clinical stages. On the other hand, patients with clinical or radiographic signs of periodontal disease or other pathologies involving the soft or hard tissues were excluded. Additional exclusion criteria included any presence of malocclusion in the lateral maxilla, such as severe crowding, excessive spacing, irregular tipping and torquing, retained deciduous teeth, or the presence of fixed orthodontic appliances. All selected patients were informed about the study protocol, and their written informed consent was obtained. For minor participants, written consent was obtained from their parents. All images were evaluated by an experienced dental professional (M.P.) in dentomaxillofacial CBCT morphometric analyses. The required sample size was determined using the G Power program 3.1.9.6, specifically employing the *t*-test family with the Wilcoxon rank test (one sample), based on an alpha level of 0.05, a statistical power of 95%, and an effect size of 0.5. Following the analysis of relevant studies, the required sample size was calculated to be 62 [30]. Based on these criteria, the final sample consisted of 62 patients (37.1% male, 62.9% female), with an average age of 29.96 ± 11.5 years. The patients were divided into four age groups (11–20 years, 21–30 years, 31–40 years, and 40+ years).

### 2.2. CBCT Imaging Device and Software Characteristics

CBCT images were obtained using an Orthophos XG 3D device (Sirona Dental Systems GmbH, Bensheim, Germany) with two recording settings: VOL1 HD (85 kV/6 mA, exposure time—14.3 s) and VOL2 HD (85 kV/10 mA, exposure time—5.0 s), with voxel sizes of 160 µm and 100 µm, respectively. The field of view for all CBCT images was set to 8 × 8 cm. Image analysis was performed using GALAXIS software v1.9.4 (Sirona Dental Systems GmbH, Bensheim, Germany).

### 2.3. Morphometric Parameters

**Figure 1:**
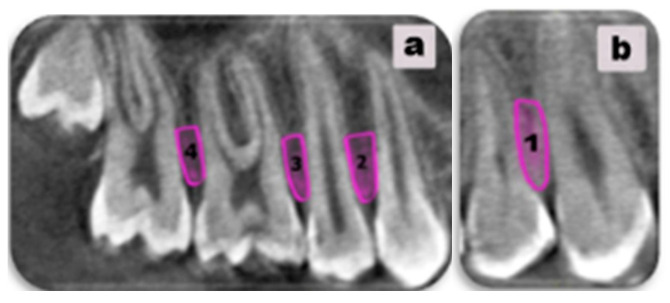
The interdental space of interest for mini-implant placement was marked. (**a**) illustrates Regions 2 (between the first and the second premolars on the right), 3 (between the second premolar and the first molar on the right), and 4 (between the first and the second molars on the right). (**b**) presents Region 1 (between the canine and first premolar).

Following the predefined criteria, the interdental space was evaluated between the following pairs of teeth in the maxillary dental arch: canine and first premolar (right: Region 1; left: Region 1′), first and second premolars (right: Region 2; left: Region 2′), second premolar and first molar (right: Region 3; left: Region 3′), and first and second molars (right: Region 4; left: Region 4′) (Figure 1).

An assessment of IPP was performed using sagittal and axial slices of the CBCT:

1. Sagittal cross-section:

Each interdental space was divided into three levels:

- Level A—4 mm from the alveolar crest;
- Level B—3 mm from the alveolar crest;
- Level C—2 mm from the alveolar crest.

**Figure 2:**
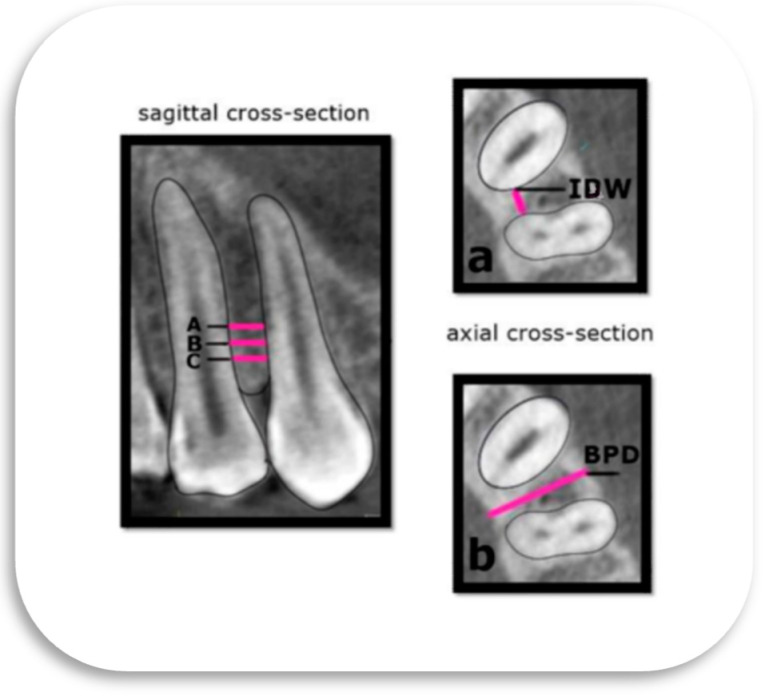
The definition of morphometric parameters of interest on CBCT images of the interdental space of the lateral segment of the maxilla. Sagittal cross-section (left): A sagittal CBCT view with the marked levels of interest. The linear measurements were made at the level of 4 mm (Level A), 3mm (Level B), and 2mm (Level C) from the alveolar crest. Axial cross-section (right): Selected morphometric parameters for analyses: (**a**) IDW—the smallest mesiodistal interdental distance; (**b**) BPD—the distance between the cortical layer of the buccal to the palatal plate, measured perpendicularly through the middle of the IDW.

Figure 2 (left) highlights the specific levels of interdental bone selected for analysis.

2. Axial cross-section:

- Buccopalatal depth (BPD)—the distance between the buccal and palatal cortical bone (Figure 2a).
- Interdental width (IDW)—the smallest mesiodistal distance between the adjacent roots (Figure 2b).

All measurements were noted at all estimated levels.

### 2.4. Relationship Between Radiological Ideal Placement Point (IPP) and Dental Structures

**Figure 3:**
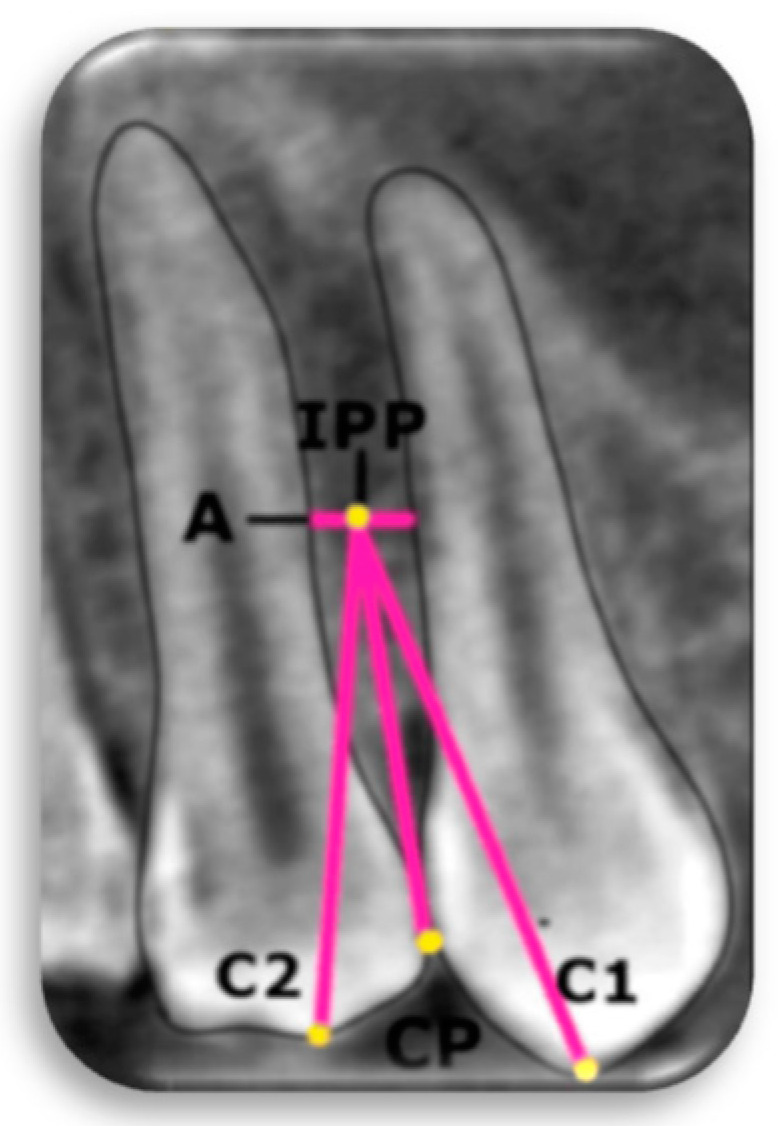
IPP—ideal placement point; A—level 4 mm from alveolar crest; C1—the cusp tips of the mesial tooth; C2—the cusp tips of the distal tooth; CP—the most coronal part of the contact surface between two teeth.

Namely, the value of 3 mm for the IDW represents the cumulative measurement accounting for both the width of the mini-implant and the required distance from the adjacent anatomical structures. Following the evaluation of the BPD and the IDW, the subsequent step involved determining the IPP. This was established based on safe zones for MI placement, as described by Poggio et al. [30] and defined as the midpoint at the evaluated level. In addition, measurements were conducted on the sagittal plane of the CBCT from the IPP to the cusp tips of the mesial tooth (C1) and distal tooth (C2), as well as from the IPP to the contact point (CP) between two adjacent teeth. In Region 4, C1 was identified as the distobuccal cusp of the first molar, while in Regions 3 and 4, C2 was defined as the mesiobuccal cusp of the adjacent molar (Figure 3).

**Figure 4:**
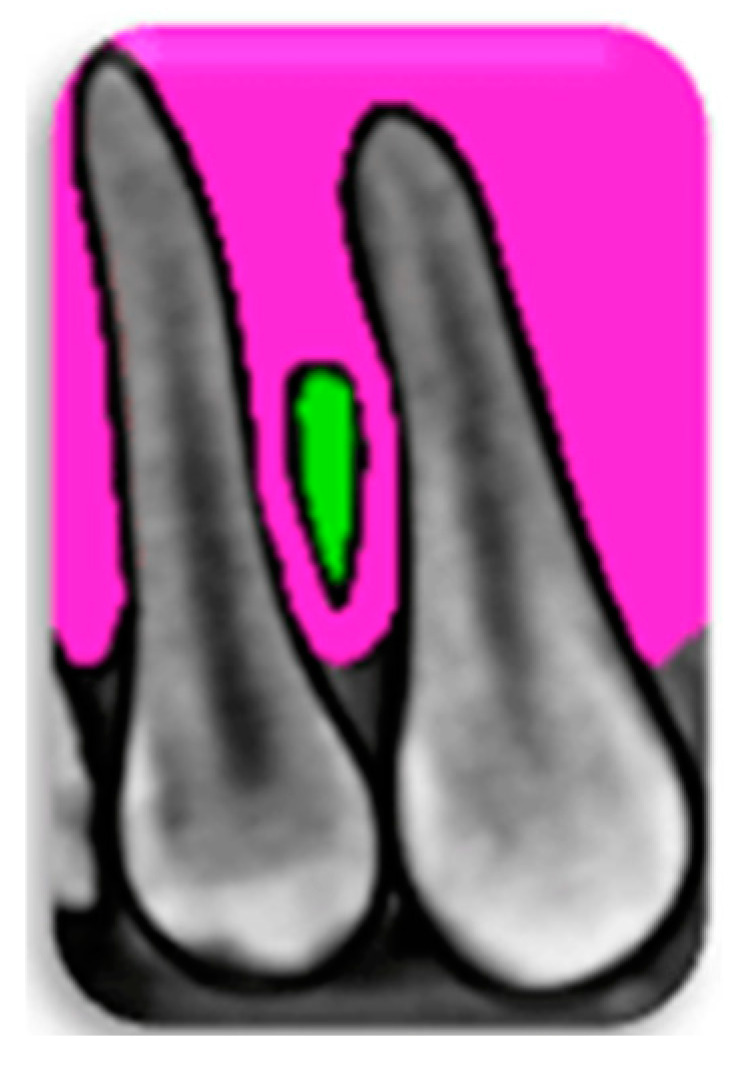
An illustration of the safe zone for MI placement, which is highlighted in green.

Figure 4 presents the safe interdental zone for MI placement based on the measurements performed.

### 2.5. Statistical Analysis

**Figure 5:**
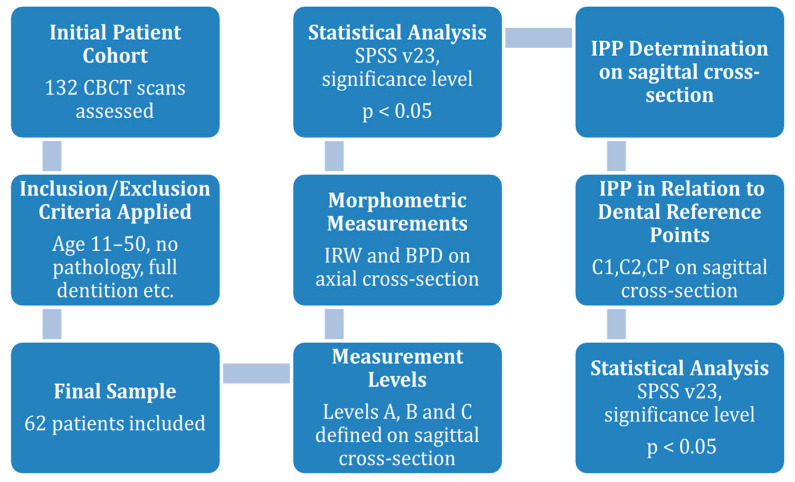
The methodological workflow of the study.

A descriptive analysis of continuous variables was performed using measures of central tendency, including the mean, the standard deviation, the minimum and maximum values, and a 95% confidence interval for the mean. Categorical variables were presented as proportions or percentages. The normality of continuous variables was assessed using the Kolmogorov–Smirnov test. Based on the results of the normality test, appropriate non-parametric (Mann–Whitney, Kruskal–Wallis) or parametric (Independent *t*-test, One-way ANOVA) tests were applied. Pearson’s correlation coefficient was used to examine relationships between continuous variables. A *p*-value of less than 0.05 was considered statistically significant. Statistical analyses were performed using the Statistical Package for the Social Sciences (SPSS) software, version 23 (Figure 5).

## 3. Results

Three-dimensional scans of 132 patients were analyzed, and following the established inclusion criteria, 62 scans were selected for further evaluation.

### 3.1. Demographics and Group Classification

Out of the selected 62 patients who met all the inclusion criteria for participation in this study, 23 were male (37.1%) and 39 were female (62.9%). The selected cohort was considered representative for evaluating the targeted clinical outcomes across both genders. The mean age of the study participants was 29.96 ± 11.50 years. The patients were categorized into age groups, as presented in Table 1.

### 3.2. Statistical Differences Between the Groups

Significant differences between the age groups were found for various parameters, including the buccopalatal depth (BPD) in Region 2 (Levels A, B, and C), Region 3 (Levels A, B, and C), and Region 4 (Level C). Additionally, significant differences were observed in the C1-IPP length in Region 1 and the CP–IPP length in Regions 2′ and 4′. Differences between genders were observed in Region 4′ (Levels A and B), as well as in the CP–IPP length in Region 3. The statistically significant findings, including those highlighting intergroup differences, are detailed in Table 2 and Table 3.

### 3.3. Relationship Between Interdental Width and Buccopalatal Depth

A statistically significant positive correlation was found between the IDW and BPD at Levels A, B, and C in Region 1. In contrast, a statistically significant negative correlation was observed between the IDW and BPD at Level C in Region 2′.

### 3.4. Descriptive Data on Mean Interdental Width and Buccopalatal Depth

Table 4 presents the mean interdental width measured at each level on the right and left sides. Mean values for the BPD at various levels are shown in Table 5. The highest percentage of IDW measurements exceeding 3 mm was found in Region 4 (67.7%) at Level A, while Region 2′ exhibited the lowest percentage of IDW exceeding 3 mm (33.9%) at the same level, as shown in Table 6.

### 3.5. Correlation Analysis

A statistically significant positive correlation was found between the left and right quadrants for the IDW and BPD in Region 1-1′ and Region 2-2′, as well as CP-IPP lengths in Region 3-3′. A positive correlation was also observed between the left and right C1–IPP and C2-IPP lengths, as shown in Table 7.

## 4. Discussion

Namely, as previously mentioned, the correct insertion of TADs requires sufficient bone availability. The interdental bone often presents limited space between roots, which can complicate the placement of TADs [9]. However, complications such as root perforation can be prevented through appropriate radiological assessment. In this context, CBCT has been established as the gold standard in dentofacial imaging and enables precise morphometric analysis [16].

At the beginning of our study, we analyzed morphometric parameters according to age and gender distribution (Table 2 and Table 3) at different estimated levels. As shown in the results, we observed statistically significant differences between males and females for measurements such as the BPD and CP-IPP. On the other hand, a study by Deguchi et al. did not report any gender-related differences [11]. In addition, we identified age-related differences, whereas Deguchi et al. reported that age had no impact on the evaluated morphometric parameters, such as the BPD and CP-IPP [11]. In contrast, Dumitrescu et al. [42] reported that older patients in Group 4 presented a reduction in the BPD compared to younger individuals, which is consistent with the findings of our study. This may have clinical relevance when planning TAD insertion in older patients, particularly in the region between the upper premolars, where smaller dimensions of MIs should be considered to ensure safe and effective placement [43].

Table 4 presents the IDW analysis. As previously mentioned, the highest mean IDW value at Level A was observed in Region 3 (3.2 mm), while the lowest IDW value was noted in Region 4′ (2.37 mm). Similar findings have been reported in numerous studies [33,44,45]. The results of our study align with those of Lee et al. [46], who reported sufficient mesiodistal space greater than 3 mm at Level A in the posterior maxilla. Likewise, Poggio et al. [30] reported the highest IDW values at 5 mm from the alveolar crest between the first and second premolars and between the canine and first premolar (3.5 mm and 4.3 mm, respectively). In contrast, they observed the lowest IDW values between the first and second maxillary molars (2.3 mm). In the majority of cases, Region 4 exhibited a satisfactory IDW value at Level A, with 67.7% of patients having more than 3 mm of interdental space. Based on these findings, it can be suggested that the safest zones are located between the first and second molars on the right side. Conversely, the region between the first and second premolars on the left side requires more detailed morphometric assessment to avoid potential complications in areas with vulnerable bone dimensions.

As shown in Table 5, we performed a BPD analysis at all estimated levels (A, B, and C). According to the literature [16], the most commonly used TADs range in length from 6 to 12 mm and in diameter from 1 to 2 mm. From the perspective of the BPD, this information is clinically relevant. Specifically, patients with insufficient BPD values (e.g., 8.17 mm in Region 4, between the first and second molars) require special attention during TAD placement. In such cases, clinicians should consider using shorter TADs, approximately 6–8 mm in length. On the other hand, the highest BPD value was observed between the first and second molars on the right side, followed by the region between the second premolar and first molar on the left side (12.22 mm and 11.04 mm, respectively). These morphometric values allow clinicians to safely use TADs with lengths of 10–12 mm. Lee et al. [46] reported similar findings and emphasized that the safest zones in terms of the BPD are between the second premolars and first molars, as well as between the molars in the upper arch. Our findings are also in agreement with those of Purmal et al. [16].

In cases in which the IDW is insufficient and TAD insertion is planned at the mucogingival junction (5 mm from the alveolar crest), there is a higher risk of reduced stability, an increased incidence of TAD loss, and chronic soft tissue inflammation [9,47]. Therefore, modified screw heads in the region between the upper molars [48,49], variations in thread design [5,50], or adjusting the insertion angle may offer additional anchorage [51,52].

The final section of our study highlights the importance of landmarks located on dental structures (cusps and contact points). An analysis of the evaluated parameters (C1-IPP, C2-IPP, and CP-IPP) revealed no statistically significant difference between the measurements obtained on the left and right sides. Our research showed that the average values for C1-IPP and C2-IPP were 12.81 mm and 11.98 mm (Table 8). The highest mean value for CP-IPP length was in Region 3′ (10.03 mm). On the other hand, CP-IPP in Region 4′ had the lowest mean value (9.42 mm) (Table 8).

Based on the current public data, we did not find studies using a similar methodological approach for morphometric evaluation. We chose to analyze these specific points to contribute to the existing literature on the anatomical characteristics of this region. Furthermore, this methodological approach may provide clinicians with better insight into the anatomical complexity of the maxillary interdental area. The use of three reference points for linear measurements offers greater detail in identifying optimal TAD placement sites, making the procedure more predictable.

Despite the clinical relevance of the findings, several limitations must be acknowledged. The sample consisted exclusively of individuals from a Serbian cohort, which may limit the generalizability of the results to other ethnic populations. In addition, while the radiological measurements were comprehensive, this study did not include clinical validation through actual implant placement and follow-up, making it difficult to assess how reliably the proposed insertion sites translate into successful clinical outcomes. Future multi-center studies involving more diverse populations, as well as clinical trials that evaluate the performance of mini-implants inserted at CBCT-determined ideal points, are recommended to confirm and expand upon these findings.

## 5. Conclusions

In summary, the findings of this study suggest that morphological analysis can help identify significant correlations between various dental structures and interdental bone characteristics, such as the interdental width (IDW) and the buccal bone depth (BPD). Although this approach can serve as a valuable reference for clinicians during TAD placement planning, it does not eliminate the necessity for individualized radiographic assessment using CBCT. Notably, certain radiological landmarks, such as the cusp tip, are clinically visible, offering potential reference markers for intraoral evaluations. Future research should further investigate the use of such landmarks in clinical settings and explore their reliability in guiding intraoral measurements and implant planning.

## Figures and Tables

**Table 1:** Patients divided into groups by age.

Age Group				
	Frequency	Percent	Valid Percent	Cumulative Percent
11–20	17	27.4	27.4	27.4
21–30	16	25.8	25.8	53.2
31–40	13	21.0	21.0	74.2
41+	16	25.8	25.8	100.0
Total	62	100.0	100.0	

**Table 2:** A descriptive analysis of the measurements that showed statistical significance according to the age group.

Age Groups		N	Mean	Std. Deviation	Std. Error	95% Confidence Interval for Mean		Minimum	Maximum	Sig. (*p* Values)
						Lower Bound	Upper Bound			
Region 2 BPD Level A	11–20	17	9.87	1.37	0.33	9.17	10.58	7.39	11.86	0.021 *, **
	21–30	16	9.31	0.70	0.17	8.93	9.68	7.93	10.24	
	31–40	13	9.33	0.61	0.17	8.96	9.70	8.65	10.94	
	41+	16	8.70	1.19	0.30	8.06	9.33	6.49	10.50	
	Total	62	9.31	1.11	0.14	9.03	9.59	6.49	11.86	
Region 2 BPD Level B	11–20	17	9.94	1.27	0.31	9.28	10.60	7.49	11.56	0.001 *, **
	21–30	16	9.26	0.70	0.18	8.89	9.64	8.06	10.45	
	31–40	13	9.09	0.47	0.13	8.81	9.37	8.42	9.99	
	41+	16	8.54	1.09	0.27	7.96	9.12	6.83	10.43	
	Total	62	9.22	1.07	0.14	8.95	9.50	6.83	11.56	
Region 2 BPD Level C	11–20	17	9.70	1.23	0.30	9.06	10.33	7.13	11.46	0.000 *, **
	21–30	16	9.08	0.71	0.18	8.70	9.46	7.96	10.23	
	31–40	13	8.65	0.76	0.21	8.19	9.11	6.97	9.91	
	41+	16	8.07	1.05	0.26	7.50	8.63	6.35	9.89	
	Total	62	8.90	1.14	0.14	8.61	9.19	6.35	11.46	
Region 3 BPD Level A	11–20	17	11.20	1.29	0.31	10.53	11.86	9.17	13.90	0.005 *, **
	21–30	16	10.34	0.60	0.15	10.02	10.65	8.84	11.26	
	31–40	13	11.07	1.00	0.28	10.47	11.67	9.78	13.53	
	41+	16	9.99	1.17	0.29	9.37	10.61	8.18	12.27	
	Total	62	10.64	1.15	0.15	10.35	10.93	8.18	13.90	
Region 3 BPD Level B	11–20	17	11.44	1.27	0.31	10.79	12.09	9.04	13.88	0.005 *, **
	21–30	16	10.47	0.62	0.15	10.14	10.80	8.89	11.50	
	31–40	13	11.35	1.07	0.30	10.70	12.00	10.05	13.50	
	41+	16	10.33	1.11	0.28	9.74	10.92	8.28	12.51	
	Total	62	10.89	1.14	0.15	10.60	11.17	8.28	13.88	
Region 3 BPD Level C	11–20	17	10.66	1.27	0.31	10.01	11.31	8.67	13.94	0.006 *, **
	21–30	16	10.16	0.74	0.19	9.77	10.55	8.14	11.12	
	31–40	13	10.79	1.05	0.29	10.16	11.43	9.35	13.31	
	41+	16	9.37	1.51	0.38	8.57	10.17	7.17	11.76	
	Total	62	10.23	1.28	0.16	9.90	10.55	7.17	13.94	
Region 4 BPD Level C	11–20	17	12.13	0.73	0.18	11.75	12.50	10.52	13.11	0.037 ”, **
	21–30	16	11.75	1.41	0.35	11.00	12.50	7.22	13.68	
	31–40	13	11.48	2.09	0.58	10.22	12.75	7.34	14.09	
	41+	16	10.89	1.48	0.37	10.10	11.69	6.33	12.46	
	Total	62	11.58	1.50	0.19	11.20	11.96	6.33	14.09	
Region 1 C1-IPP	11–20	17	13.40	1.05	0.25	12.87	13.94	11.44	15.09	0.002 *, **
	21–30	16	13.06	0.78	0.20	12.65	13.50	11.85	14.26	
	31–40	13	14.51	1.31	0.36	13.72	15.30	11.71	16.10	
	41+	16	13.47	0.75	0.19	13.07	13.87	12.42	15.03	
	Total	62	13.57	1.09	0.14	13.29	13.84	11.44	16.10	
Region 2′ CP-IPP	11–20	17	9.16	0.68	0.17	8.81	9.51	8.02	10.16	0.016 ”, **
	21–30	16	9.67	0.96	0.24	9.16	10.18	7.67	11.39	
	31–40	13	9.88	0.85	0.24	9.36	10.39	7.57	11.39	
	41+	16	9.48	0.87	0.22	9.02	9.95	7.87	11.18	
	Total	62	9.53	0.87	0.11	9.31	9.74	7.57	11.39	
Region 4′ CP-IPP	11–20	17	8.91	0.82	0.20	8.49	9.33	7.65	10.45	0.048 *, **
	21–30	16	9.62	0.91	0.23	9.14	10.11	8.10	12.01	
	31–40	13	9.93	1.35	0.37	9.12	10.74	7.30	12.01	
	41+	16	9.35	0.96	0.24	8.84	9.87	8.27	12.01	
	Total	62	9.42	1.05	0.13	9.16	9.69	7.30	12.01	

* ANOVA—statistical significance. ” KRUSKAL–WALLIS—statistical significance. ** *p* < 0.05.

**Table 3:** Descriptive analysis of measurements that showed statistical significance according to gender.

Gender		N	Mean	Std. Deviation	Std. Error	95% Confidence Interval for Mean		Minimum	Maximum	Sig. (*p* Values)
						Lower Bound	Upper Bound			
Region 4′ BPD Level A	male	23	8.96	1.17	0.25	8.45	9.47	6.99	12.80	0.028 *, **
	female	39	8.47	0.99	0.16	8.15	8.79	5.38	10.20	
	Total	62	8.65	1.08	0.14	8.38	8.93	5.38	12.80	
Region 4′ BPD Level B	male	23	8.64	1.08	0.23	8.17	9.11	6.56	11.60	0.040 *, **
	female	39	8.36	1.05	0.17	8.02	8.71	5.11	9.85	
	Total	62	8.47	1.06	0.14	8.19	8.74	5.11	11.60	
Region 3 CP-IPP	male	22	9.85	1.15	0.25	9.34	10.36	8.32	12.11	0.050 *, **
	female	40	9.31	0.91	0.14	9.02	9.61	8.17	12.84	
	Total	62	9.50	1.03	0.13	9.24	9.76	8.17	12.84	

* MANN–WHITNEY—statistical significance. ** *p* < 0.05.

**Table 4:** The mean interdental width measured at each level on the right and the left side. The satisfactory measurements (>3 mm) are marked.

Interdental Width (mm)									
		Canine and First Premolar		First and Second Premolars		Second Premolar and First Molar		First and Second Molars	
	Level	Mean	SD	Mean	SD	Mean	SD	Mean	SD
Right									
	4mm(A)	3.13	0.78	3.05	0.70	3.21	0.84	3.18	0.79
	3mm(B)	2.92	0.77	2.78	0.68	2.93	0.78	2.94	0.71
	2mm(C)	2.70	0.75	2.53	0.66	2.69	0.73	2.73	0.63
Left	4mm(A)	3.08	0.67	3.17	0.80	3.08	0.77	2.75	0.87
	3mm(B)	2.84	0.68	2.91	0.80	2.79	0.68	2.56	0.73
	2mm(C)	2.59	0.65	2.77	1.35	2.53	0.59	2.37	0.61

**Table 5:** The mean buccopalatal depth measured at each level on the right and the left side.

Buccopalatal Depth (mm)									
		Canine and First Premolar		First and Second Premolars		Second Premolar and First Molar		First and Second Molars	
	Level	Mean	SD	Mean	SD	Mean	SD	Mean	SD
Right									
	4mm(A)	9.05	0.99	9.31	1.10	10.64	1.15	11.93	1.50
	3mm(B)	8.75	1.21	9.22	1.07	10.88	1.14	12.22	1.51
	2mm(C)	8.39	1.19	8.90	1.13	10.23	1.28	11.58	1.50
Left	4mm(A)	9.01	1.44	9.55	1.25	10.77	0.95	8.65	1.08
	3mm(B)	8.79	1.37	9.28	1.46	11.04	0.98	8.46	1.06
	2mm(C)	8.26	1.49	8.89	1.83	10.45	0.94	8.17	1.07

**Table 6:** Percentage of patients with IDW over 3 mm at each level.

Region	Level	IDW over 3 mm in %
1	A	54.80
	B	46.80
	C	37.10
2	A	56.50
	B	41.90
	C	24.20
3	A	59.70
	B	51.60
	C	33.90
4	A	67.70
	B	43.50
	C	32.30
1′	A	66.10
	B	43.50
	C	33.90
2′	A	61.30
	B	46.80
	C	29.00
3′	A	53.20
	B	33.90
	C	25.80
4′	A	33.90
	B	19.40
	C	14.50

**Table 7:** A statistically significant positive correlation between the left and right C1-IPP and C2-IPP lengths.

		Pearson Correlation	*p*-Value
Region 1-1′	C1-IPP	0.28	0.027
	C2-IPP	0.39	0.002
Region 3-3′	C1-IPP	0.53	0.000
	C2-IPP	0.29	0.025
Region 4-4′	C1-IPP	0.33	0.009
	C2-IPP	0.38	0.002

**Table 8:** Mean values with Standard deviations for C1-IPP, C2-IPP, and CP-IPP lengths for each region.

	Length	Mean Values (mm)	SD
Region 1	C1-IPP	13.56	1.08
	C2-IPP	12.40	0.88
	CP-IPP	9.76	1.14
Region 2	C1-IPP	12.63	0.83
	C2-IPP	12.19	0.77
	CP-IPP	9.55	0.96
Region 3	C1-IPP	12.45	1.13
	C2-IPP	11.84	1.28
	CP-IPP	9.50	1.02
Region 4	C1-IPP	12.76	1.31
	C2-IPP	11.57	1.07
	CP-IPP	9.59	0.71
Region 1′	C1-IPP	13.85	1.07
	C2-IPP	12.71	0.87
	CP-IPP	9.71	0.87
Region 2′	C1-IPP	12.54	0.75
	C2-IPP	11.98	0.83
	CP-IPP	9.52	0.86
Region 3′	C1-IPP	12.55	1.09
	C2-IPP	11.98	1.15
	CP-IPP	10.03	1.16
Region 4′	C1-IPP	12.43	0.94
	C2-IPP	11.24	0.76
	CP-IPP	9.42	1.05

## Data Availability

Data available upon request from the authors.
